# Influence of Maternal Aging on Mitochondrial Heterogeneity, Inheritance, and Function in Oocytes and Preimplantation Embryos

**DOI:** 10.3390/genes9050265

**Published:** 2018-05-21

**Authors:** Dori C. Woods, Konstantin Khrapko, Jonathan L. Tilly

**Affiliations:** Laboratory for Aging and Infertility Research, Department of Biology, Northeastern University, Boston, MA 02115, USA; k.khrapko@northeastern.edu

**Keywords:** mitochondria, mitochondrial DNA, oocytes, embryos, inner cell mass, trophectoderm, aging

## Abstract

Contrasting the equal contribution of nuclear genetic material from maternal and paternal sources to offspring, passage of mitochondria, and thus mitochondrial DNA (mtDNA), is uniparental through the egg. Since mitochondria in eggs are ancestral to all somatic mitochondria of the next generation and to all cells of future generations, oocytes must prepare for the high energetic demands of maturation, fertilization and embryogenesis while simultaneously ensuring that their mitochondrial genomes are inherited in an undamaged state. Although significant effort has been made to understand how the mtDNA bottleneck and purifying selection act coordinately to prevent silent and unchecked spreading of invisible mtDNA mutations through the female germ line across successive generations, it is unknown if and how somatic cells of the immediate next generation are spared from inheritance of detrimental mtDNA molecules. Here, we review unique aspects of mitochondrial activity and segregation in eggs and early embryos, and how these events play into embryonic developmental competency in the face of advancing maternal age.

## 1. Introduction

For over 50 years, scientists have pondered and experimentally pursued the influence of organismal aging on mitochondrial function, and of mitochondrial dysfunction on organismal aging, in a wide variety of species and cell types [1,2,3,4,5,6,7,8]. Although the cause–effect relationship between changes in mitochondrial function and aging remains unsettled, many studies have concluded that derangements in one or more aspects of mitochondrial biology probably contribute to the manifestation of aging-related phenotypes in cells [2,3,4,5,6,7,8]. Much of this work has historically been rooted in basic principles of mitochondrial bioenergetics, such as increased oxidative damage to macromolecules by reactive oxygen species (ROS) released as a by-product of ATP synthesis or progressive loss of cellular homeostasis associated with deficits in ATP-generating capacity, in the context of cellular aging. However, several other fundamental features of mitochondrial biology have been comparatively understudied in aging. One such example is the diversity of mitochondrial functions not directly related to the production of ATP. For example, mitochondria directly participate in management of intracellular ion gradients, particularly those involving release of intracellular Ca^2+^ stores, that can influence a wide variety of gene expression pathways and signal transduction cascades [9]. Mitochondria can also actively synthesize bioactive factors, such as melatonin [10], which then affect cellular metabolism and function. A second example is the diversity in mitochondrial subtypes, which we refer to as mitochondrial heterogeneity, found in all cells. Although it has been recognized for years that mitochondria vary widely in many parameters, such as size, ultrastructure and degree of membrane polarization (mitochondrial membrane potential, Δψ_m_), the possibility that distinct subpopulations of mitochondria within the same cell control or modify different processes in a subtype-specific manner remains untested. One last example is the diversity in the hundreds of nuclear-encoded gene products that are differentially trafficked to mitochondria, and post-translationally regulated, in a cell lineage-specific manner [11,12,13]. This variation in the mitochondrial proteomic landscape across cell types, coupled with numerous reports that mitochondria can in turn communicate back to the nucleus via a form of retrograde signaling [14,15,16,17], is an exciting, but still underexplored, endpoint for studies of how phenotypic and functional changes in cells associated with aging could be mediated through alterations in mitochondria or, more specifically, subtypes of mitochondria.

To more fully address these key gaps in knowledge, the female germ line has emerged as a uniquely powerful model system to study the relationships between aging and mitochondrial biology for several reasons. Perhaps the most important of these is that transgenerational passage of mitochondria, and the genomic material these organelles contain (mitochondrial DNA (mtDNA)), is strictly uniparental through the egg, with paternal mitochondria rapidly degraded after sperm penetration during fertilization [18,19,20,21,22,23,24]. Female germ cells therefore bear sole responsibility for ensuring that mtDNA integrity is preserved for inheritance through each successive generation. Tremendous effort has been expended to understand exactly how the female germ line is able to accomplish this arduous task. One of the major obstacles faced by eggs in doing this is the need to balance the high bioenergetic demands of achieving embryonic developmental competency against the mutagenic effects of ROS, generated locally in mitochondria during ATP synthesis, on DNA contained within mitochondria of eggs and embryos. Successful inheritance of clean mtDNA through the egg also seems counterintuitive to the fact that mitochondria in the female germ line undergo a two-to-four log-order amplification in numbers as primordial germ cells (PGCs) progressively differentiate into oocytes and then into mature eggs [25,26,27], in context with mtDNA suffering an estimated three-log-order higher mutation rate than nuclear DNA per duplication cycle [28]. These observations, when considered with the fact that, irrespective of the mutational driver (oxidative damage or duplication errors), mtDNA is not subject to the same high degree of surveillance, damage detection and repair that exists for nuclear DNA, indicate that female germ cells face a unique set of mitochondrial-related challenges in meeting their ultimate purpose.

Another advantage of using the female germ line for studies of aging and mitochondrial dysfunction is that, in many species, female reproductive (ovarian) aging and infertility occur long before animals are considered chronologically aged [29,30,31,32,33,34]. Because the ovaries undergo this relatively early aging process, endpoint analyses of this organ, and the cells it contains, are less apt to be influenced by undefined extrinsic or systemic consequences of general organismal aging. With that said, maternal age is widely considered the single most important variable which influences pregnancy success rates. In turn, oocyte donation studies have demonstrated that the deterioration of egg and embryo quality, rather than a more generalized inability of the aging female reproductive system to initiate and sustain a successful gestation, is the primary driver of aging-associated loss of fertile potential [35,36]. Although many parameters have been used over the years to define changes in egg quality when assessing the impact of maternal aging on reproductive success, abnormalities in mitochondrial function and chromosomal segregation (aberrant meiotic spindle formation and aneuploidy) are the most frequently cited causes of developmental incompetency and embryonic failure [37,38,39,40,41,42,43,44,45,46,47,48]. Furthermore, interventions that target pathways tied to either maintenance or restoration of mitochondrial homeostasis in eggs result in dramatic improvements in spindle formation, chromosomal dynamics and fertility outcomes [45,49,50,51,52].

These studies have consistently pointed to the ability of female germ cells to correctly align and segregate chromosomes during meiosis as an aging-sensitive process highly dependent on mitochondrial function. For example, stepwise institution of moderate dietary caloric restriction (CR) in female mice during adult life completely prevents the aging-associated increases in meiotic spindle formation abnormalities and aneuploidy in eggs [45], resulting in a dramatic extension of reproductive lifespan well into advanced chronological ages [50]. Similar improvements in egg quality with age can be achieved in mice through targeted disruption of the gene encoding peroxisome proliferator-activated receptor γ coactivator-1α (PGC-1α) [45], a protein widely known for its central role in regulating mitochondrial biogenesis [53,54], as well as through chronic treatment with either resveratrol (trans-3,5,4′-trihydroxystilbene) [55] or coenzyme Q10 (coQ10) [56], both of which have been touted as mitochondrial nutrients and potential anti-aging therapies [51,57,58]. With this information as a preface, here we overview the current state of knowledge regarding several unique aspects of studying mitochondria in the female germ line, with a principal focus on the impact of maternal aging on mitochondrial dynamics in oocytes and embryos. Areas where significant gaps in knowledge exist are also highlighted, with potential hypotheses and experimental approaches offered for future testing.

## 2. The Germline Mitochondrial DNA Bottleneck and Purifying Selection

Experimental delineation of the mechanism by which mtDNA is passed through the female germ line in a relatively pristine state generation after generation has eluded scientists for decades. Indeed, fertilized eggs need to accomplish what seems to be, based on the discussions above (see Section 1), a near-impossible feat. One could argue that oocytes and eggs somehow shield their mtDNA from mutations and damage, thereby allowing transmission of only pristine mitochondrial genomes to offspring; however, this is discordant with findings that eggs have significant numbers of mtDNA mutations and deletions that become more prevalent with reproductive age [59,60,61]. Compounding the issue, an increasing mtDNA mutational load is only part of a much bigger challenge for the female germ line since each egg contains hundreds of thousands of mtDNA molecules. While a random mutation in any one of these molecules would have no physiological consequences, progressive accumulation of these invisible mutations over many generations would eventually be disastrous to species survival. Two general mechanisms, working in tandem, have been offered to best explain how organisms deal with this potentially catastrophic issue: the mtDNA bottleneck and purifying selection [62,63,64,65,66,67,68,69,70,71]. In its most simplistic form, the mtDNA bottleneck is thought to work by accelerating genetic drift in the intracellular populations of mitochondria to increase the variance in mutational load across an entire population of cells. In those cells of the population where the highest load of allocated mutations exceeds a physiological threshold, an as-yet undefined phenotypic or functional change then triggers purifying selection to remove those cells enriched with detrimental mtDNA genotypes. In females, it is believed that germline purifying selection works after the germline mtDNA bottleneck to remove those germ cells that receive a larger proportion of mutant mtDNA molecules during development. Germ cells with less severe mutational loads survive the bottleneck, and some of these are then used to form eggs that propagate the species. Without this mechanism in place, silent and unchecked spreading of multiple invisible mtDNA mutations would accumulate each generation until the population becomes irreversibly doomed because of mtDNA meltdown.

As fundamentally critical as the germline mtDNA bottleneck appears to be to species survival, how the bottleneck works remains obscure and a matter of debate. Several different hypothetical models have been proposed over the years [71,72], but none to date have been experimentally validated. The original model postulated that embryonic PGCs contain very few mitochondrial genomes per cell (~1 × 10^2^) so that genetic drift in the intracellular mitochondrial population during many rounds of PGC proliferation would result in partial or complete segregation of the more detrimental mitochondrial genotypes into some of the PGCs [63]. One study has estimated that the mtDNA bottleneck in humans needs to contain as few as 30 mtDNA molecules (and no more) to account for the observed distribution of mitochondrial genotypes [69]. Such explanations appear reasonable; however, several groups have reported that the number of mtDNA copies in PGCs is much higher, ranging 1–2 × 10^3^ per cell [73]. As such, a simple genetic drift mechanism is unlikely to explain the observed mtDNA bottleneck effect.

This prompted the formulation of several alternative bottleneck models seeking to reduce the number of segregating units despite the apparently large number of mtDNA molecules [66,67,71,72,73,74,75]. Further complicating this, recent work has attributed mtDNA bottleneck positions to widely different stages in germline development. Contrasting the original model that postulates the mtDNA bottleneck operates prenatally in PGCs [63,72], a different model places the mtDNA bottleneck postnatally during oocyte growth [66]. Another study has placed the mtDNA bottleneck into the early preimplantation embryo [76]. While these models were proposed years ago, it is still not clear which model, or combination of models, is correct [71]. A major barrier to uncovering the precise mechanism of the mtDNA bottleneck is that it is usually characterized by one parameter: the amount of variance in the levels of heteroplasmy among germ cells. Unfortunately, this single parameter carries very limited information. Future studies designed to construct entire mtDNA phylogenetic trees, unburdened by current limitations in sequencing technologies that introduce mutational artifacts arising from sequencing and PCR errors (see Section 6), will carry significantly more information and thus should allow discrimination between the alternative proposed mechanisms with a high degree of confidence.

Even if continued studies eventually identify the precise bottleneck mechanism responsible for maintenance of mtDNA integrity in the female germ line from one generation to the next, a fundamentally important question will remain: What is the post-fertilization fate of mtDNA molecules bearing high mutational burdens in eggs from a somatic point of view? In other words, are mitochondria carrying a higher proportion of mutant mtDNA molecules in eggs haphazardly transferred into the embryo proper at the blastocyst stage to be carried through into non-germ line lineages that arise out of the embryo? Conversely, are these detrimental molecules discarded somewhere along the developmental pathway of embryogenesis to lessen the mutational burden on the soma of newly formed embryos? This does not mean that the molecules in question are loaded with many mutations. Rather, keeping a pool of mtDNA molecules in pristine condition, and subsequently ensuring that molecules from that pool are the only molecules inherited, allows for a reduction in the cumulative burden of inherited mutations which occurs over the span of many generations. This question has been largely overlooked by the scientific community, but is critical to address as efforts are made to more fully understand mitochondrial inheritance. Indeed, recent studies have shown that even low levels of maternally transmitted mtDNA mutations can negatively affect both health and overall lifespan in next-generation offspring [77,78]. These observations underscore the critical need to explore the mechanisms that exist to minimize the passage of mtDNA mutations from mother, through the fertilized egg, to somatic cells of her offspring in a single generation, in addition to work focused on identification of the mechanisms that maintain mtDNA integrity in the female germ line across sequential generations. Along these lines, essentially nothing is known of the influence of maternal aging on the efficiency of the mtDNA bottleneck to cleanse germline mitochondrial genomes in preparation for transmission through eggs to each successive generation. Additionally, the possibility that eggs of females at advanced reproductive ages are more prone to aberrantly transmit detrimental mtDNA molecules into embryonic somatic lineages of next-generation offspring remains untested.

## 3. Mitochondrial Dynamics and Heterogeneity in Eggs and Embryos

**Figure 1:**
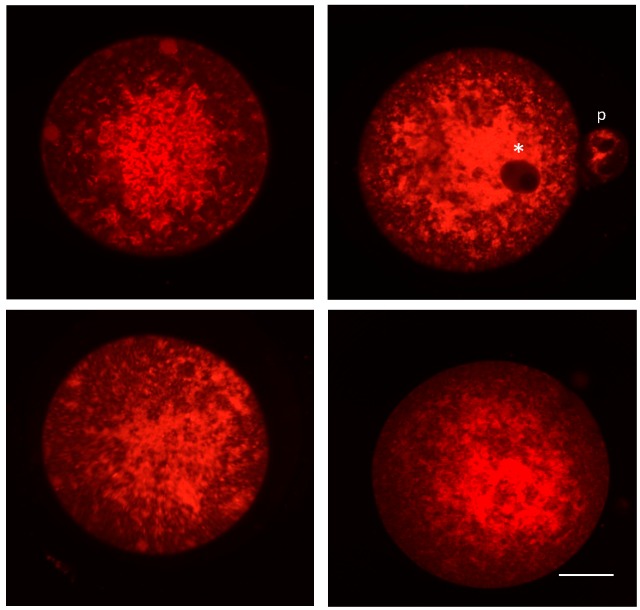
Confocal photomicrographs of mitochondrial distribution patterns in mouse oocytes. Oocytes collected from adult female mice following hormone stimulation-induced superovulation were classified as metaphase-II (extruded first polar body), and then incubated with MitoTracker Red (Thermo Fisher Scientific, Waltham, MA). Oocytes were immediately coverslipped and imaged using a Zeiss laser scanning confocal microscope. Mitochondria are noticeably punctate and present throughout the oocyte. Examples demonstrate multiple focal planes, with perinuclear distribution (indicated by white asterisk), and a polar body (p) is visible in the top right panel. Scale bar = 15 μm.

**Figure 2:**
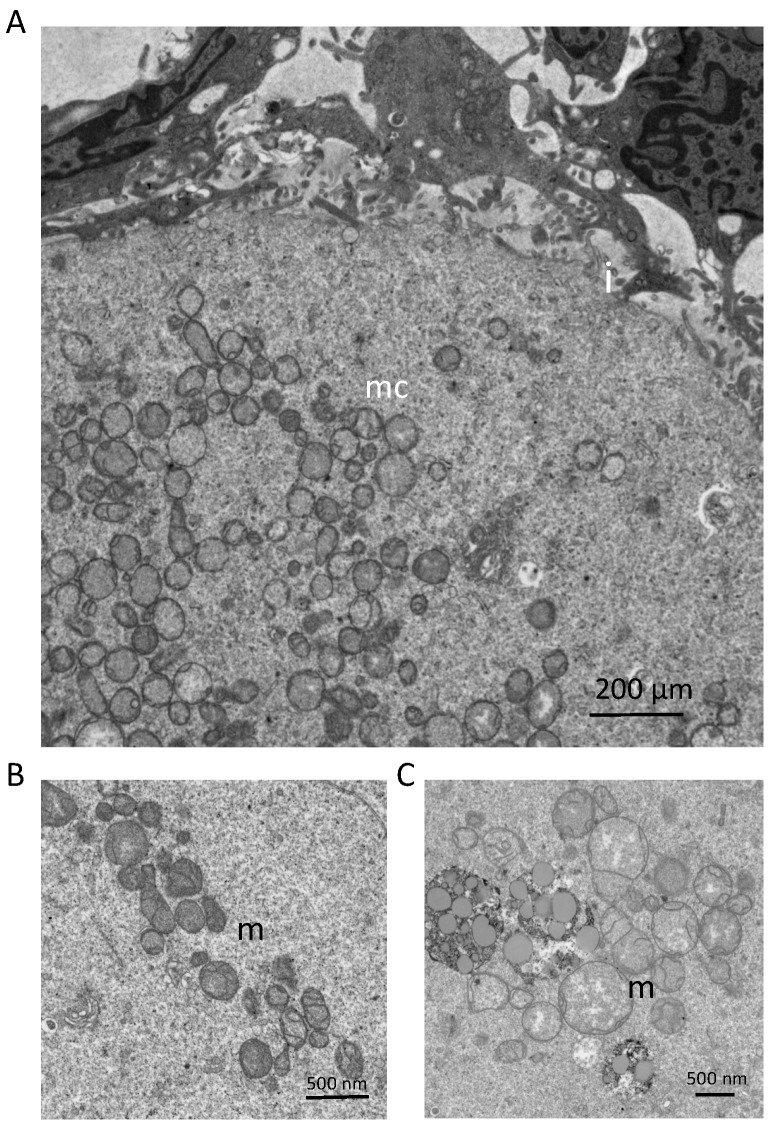
Mitochondrial ultrastructural features, as determined by transmission electron microscopy, in primary-stage oocytes contained within follicles of reproductive-age human ovaries: (**A**) Mitochondria are localized as clusters (mitochondrial clusters; mc) within oocytes. The oocyte-granulosa cell interface (i) is also depicted, and demonstrates areas of interdigitation between the oocyte and surrounding granulosa cells. Scale bar = 200 µm. (**B**,**C**) Mitochondrial morphology (m) depicted at two magnifications reveals that most mitochondria exhibit an ovoid morphology, dense matrices, and few cristae. However, a large variation in mitochondrial size can be observed, along with multiple examples of mitochondria that fall outside of the characteristic morphology described above. Tissues were collected and prepared for TEM by non-coagulative aldehyde fixation, followed by sectioning on an ultramicrotome. Images were acquired using a JEOL JEM-1010 transmission electron microscope.

A growing body of evidence strongly supports the existence of mitochondrial heterogeneity in both eggs and preimplantation embryos, as well as of a potential segregation mechanism that differentially allocates mitochondrial subtypes into the two principal embryonic lineages that are first specified during blastocyst formation: the inner cell mass (ICM; composed of primitive endoderm and pluripotent epiblast cells), which gives rise to the embryo proper, and the trophectoderm (TE; composed of extraembryonic cells), which gives rise to the placenta. For example, many studies have documented the coexistence of mitochondrial populations with low Δψ_m_ and high Δψ_m_ in individual eggs [79]. Additionally, the highly-polarized mitochondria in eggs are distributed almost exclusively in the outer pericortical domain [79,80,81], potentially reflecting a need for active mitochondria in this region of eggs to provide localized metabolic support or management of Ca^2+^ oscillations at the time of sperm penetration. This active subpopulation represents a small percentage of the total mitochondrial pool in oocytes [79], with little known of the role(s) that the remaining mitochondria, which are distributed uniformly throughout the egg (Figure 1), might play. Notably, mitochondrial heterogeneity in eggs is not restricted to differences in only membrane polarization status. Early electron microscopy studies have identified a wide spectrum of mitochondrial sizes in oocytes, with those in mouse eggs ranging from as small as 0.2 µm to as large as 0.6 µm or more [25]. Significant heterogeneity in size and ultrastructural morphology is also apparent in human oocytes (Figure 2). However, the significance, if any, of this heterogeneity in mitochondrial Δψ_m_ or size to oocyte maturation or post-fertilization embryonic developmental competency remains to be determined.

With respect to the existence of mitochondrial heterogeneity in preimplantation embryos, prior studies using mitochondrial membrane probes have reported that mitochondria with high Δψ_m_ are primarily localized to the TE whereas most mitochondria in the ICM are characterized by low Δψ_m_ [79]. Human blastocysts also show stark lineage-specific differences in the levels of cytochrome *c* oxidase activity in mitochondrial subpopulations, with very low levels in the ICM and high levels in the TE [82]. These findings align well with the results of studies based on Δψ_m_ showing that mitochondria in the TE are highly polarized, whereas mitochondria in the ICM are not [79]. Importantly, the method used to measure cytochrome *c* oxidase activity in human embryos, which involved ultrastructural analysis of mitochondrial matrix density, unveiled a striking finding that was not discussed in detail—more than 90% of the mitochondria in the ICM appeared small to intermediate size with poorly developed or no cristae, while more than 90% of the mitochondria in the TE appeared intermediate to large size with over one-third exhibiting complex well-developed cristae [82]. Since all mitochondria present in preimplantation embryos arise from the eggs that were fertilized to produce them, these data collectively indicate that mitochondrial subpopulations in eggs, distinguished by differences in size and Δψ_m_ (and, as a likely consequence, bioenergetic potential), may also exhibit markedly different post-fertilization fates during specification of the ICM and TE lineages at the blastocyst stage of embryogenesis. An open question for future studies is to determine if different mitochondrial subpopulations are somehow either marked for detection and subsequent lineage-specific segregation or, conversely, perhaps even promote lineage-specific segregation during embryogenesis.

What could be driving these apparent differences in segregation patterns? To answer this, one must first consider the two models that have been proposed thus far to explain the first cell-fate decision process that occurs during preimplantation embryonic development [83,84]. According to the positional model, the relative outer or inner location of individual blastomeres within the developing embryo determines the fate of those cells that will form the TE (outermost) or ICM (innermost). In the polarity model, differential allocation of factors based on cellular polarity (i.e., apical versus basolateral localization) occurs during asymmetric division of a blastomere in compacted eight-cell embryos, which then drives the fate of each daughter cell towards one lineage or the other based on asymmetric partitioning of factors required for acquisition of an ICM or TE identity [83,84]. Mitochondria have already been identified as key forces behind establishment of polarity in somatic cells [85,86]. Accordingly, it is reasonable to postulate that mitochondria (or perhaps a specific subtype of mitochondria) drive polarization of blastomeres during compaction of eight-cell embryos to form early morulae. Past studies have also shown that intracellular heterogeneity in Δψ_m_ reflects points of contact of a given cell with other cells [87]. In cleavage-stage mouse embryos, mitochondria with low Δψ_m_ are found predominantly at regions of intercellular contact [80]. In embryos undergoing compaction, these contact points are confined to the inward-facing or basolateral regions of blastomeres, whereas the outward-facing or apical regions are contact-free [83,84]. Thus, mitochondria with low Δψ_m_ would be clustered in the region of a blastomere that, following asymmetric division via the polarity model (producing an apical and a basolateral daughter cell), would be preferentially allocated into the innermost (basolateral) daughter cell which, according to the positional model, is destined to become ICM. As mentioned earlier, one could also postulate that a specific mitochondrial subtype preferentially segregated into an as-yet uncommitted daughter cell produced through the polarity model actively drives the fate of that cell to become ICM or TE via mitochondrial subtype-specific production of a fate-regulating factor. As discussed earlier, mitochondria are well-known to modulate many aspects of cell function aside from bioenergetics, including ion movement and production of bioactive factors; however, it is not known if these actions are generic to all mitochondria or limited to specific subtypes of mitochondria. Clearly, future studies are needed to address these possibilities.

The influence of maternal aging on mitochondrial heterogeneity in eggs and embryos has not been specifically addressed. However, various mitochondrial anomalies have been linked to the deterioration of oocyte quality as females age, and at least some these may be reflective of changes in specific mitochondrial subpopulations. The most prominent of these defects include atypical mitochondrial localization and aggregation, reduced mtDNA content, reduced Δψ_m_ (and, consequently, bioenergetic capacity), increased oxidative stress, and increased frequency of mtDNA mutations and deletions [27,34,42,45,49,51,52,56,59,60,61,88,89,90,91,92,93]. Equally important to consider are alterations in mitochondrial biogenesis and autophagy (mitophagy). During oocyte development from the immature germinal vesicle stage to the mature egg stage, a tremendous level of mitochondrial biogenesis occurs such that, by the time of fertilization, a single egg contains hundreds of thousands of mitochondria. This is critical to ensure that the developing embryo is provided with sufficient numbers of mitochondria required to sustain embryogenesis through successive cleavage divisions to the preimplantation blastocyst stage, since mitochondrial replication in embryos does not resume until after implantation [94,95].

This accelerated level of mitochondrial biogenesis during oocyte maturation is further enhanced by an apparent absence of mitophagy in oocytes [96], the latter of which can significantly alter preimplantation embryogenesis. For example, using oocyte-specific autophagy-related *5* (*Atg5*) gene knockout mice, it has been reported that mitophagy does not naturally occur in oocytes, but becomes critical after fertilization since *Atg*-null oocytes fertilized with *Atg*-null sperm arrest at the 4–8-cell stage of preimplantation embryonic development [97]. Moreover, pharmacologic induction of mitophagy in wild type eggs by rapamycin reduces fertilization rates and post-fertilization embryonic developmental potential [98]. It has also been shown that mitochondria in oocytes do not associate with autophagosomes following chemically-induced membrane depolarization [96]. The inability of oocytes to respond to mitochondrial damage and dysfunction by elevating mitophagy is underscored by data demonstrating that mtDNA copy numbers increase following exposure of oocytes to antimycin-A to disrupt mitochondrial function. In addition, the common 4977 bp deletion (ΔmtDNA 4977), which is widely used as an mtDNA mutational marker in somatic cells, accrues in oocytes of women older than 35 years of age [61].

Perhaps not surprisingly, impaired mitochondrial biogenesis has been directly implicated as a significant factor underlying poor oocyte quality associated with aging. Studies of human oocytes retrieved from women over 40 years of age revealed that mtDNA content was significantly reduced when compared to mtDNA content in eggs obtained from younger women [92]. Additionally, failure to fertilize and ovarian insufficiency have been linked to reduced mtDNA copy numbers [88,89,90]. In mouse and hamster oocytes, total mitochondrial numbers, as assessed by electron microscopy, decline with maternal age, and this occurs concomitant with reduced mtDNA content and ATP levels [93] It is important to bear in mind, however, that mtDNA content is an indirect measure of mitochondrial quantity, as an individual mitochondrion can contain 1–10 mtDNA molecules. It has been estimated that oocyte mitochondria, which in mice generally range in size 0.3–0.6 µm and harbor 1–2 copies of mtDNA per organelle [25], but direct validation of this has not been performed. Therefore, when assessing the relationship of mtDNA copy number to mitochondrial number, each variable should be considered as an independent biomarker for oocyte and embryo quality. Nonetheless, these findings collectively support the notion that advancing maternal age is tightly linked to a progressive deficiency in mitochondrial biogenesis in oocytes.

In a comprehensive clinical study of human cleavage-stage embryos (blastomeres) and blastocysts obtained from two patient cohorts of women undergoing in-vitro fertilization (IVF)—a group of women up to 37 years of age and a group of women 38 years and older—it was determined that blastomeres collected from women in the younger age cohort had significantly higher levels of mtDNA when compared with blastomeres of the older cohort [99]. This is consistent with the previous work of others, providing additional evidence of an inverse relationship between maternal age and mtDNA content in oocytes and early cleavage-stage embryos [88,89,90,91,92,100] However, dramatically different results were obtained after parallel analysis of blastocyst-stage embryos. Independent of chromosomal status, blastocysts from women aged 38 years and over had significantly higher mtDNA content versus blastocysts obtained from women who were 37 years of age or younger. Moreover, mtDNA content was found to be inversely correlated with implantation outcomes, in that not a single euploid blastocyst with elevated mtDNA copy numbers was observed to implant; by comparison, 59% of euploid blastocysts with mtDNA content within the normal range implanted successfully. Notably, irrespective of maternal age, blastocysts characterized as chromosomally abnormal contained elevated mtDNA copy numbers, indicating that aneuploidy and maternal age are independently associated with alterations in mtDNA levels [99]. These observations have been reaffirmed in blinded clinical studies, which tracked pregnancy outcomes using blastocysts from which mtDNA content was quantified prior to embryo transfer [101,102]. Although the age-associated mechanisms that underpin the increase in mtDNA content in blastocysts have not been experimentally addressed, it is possible that this is symptomatic of either defective mitophagy or elevated stress. The latter would result in greater energy demands, which would conflict with the quiet embryo hypothesis proposed by Leese, in which a low (quiet) metabolic level conveys a protective effect in developing embryos [103].

## 4. Boosting Egg and Embryo Quality through Exogenous Mitochondrial Transfer

Results from both animal and clinical studies indicate that there may be inherent properties of mitochondria that, after injection into eggs or zygotes, can directly benefit early embryonic development leading to significant improvements in pregnancy success rates. In the 1990s, a series of clinical studies termed ooplasmic transfer showed that transfer of cytoplasm from donor eggs of younger women could improve the developmental competency of eggs from women with a history of repeated IVF failure [104,105,106,107,108,109,110,111]. Although eventually halted by the United States Food and Drug Administration (FDA, Silver Spring, MD, USA) based on their views that injection of heterologous (non-patient matched) mitochondria into a woman’s egg at fertilization represented a form of foreign gene transfer for the purposes of human reproduction [112], interest in the concept that mitochondrial supplementation could benefit IVF outcomes continued through investigations in animal models [113,114]. Several years ago, a new technology that maintained the underlying principle behind heterologous ooplasmic transfer (viz. provide a boost of female germline mitochondria to developmentally compromised eggs), while simultaneously shedding the FDA’s concerns through use of autologous mitochondria, was developed and eventually entered clinical study. The premise of this new technology, termed autologous germline mitochondrial energy transfer (AUGMENT), was to use an IVF patient’s own oocyte precursor cells (referred to as oogonial stem cells (OSCs)) present in her ovaries as a matched, but more pristine, source of female germline mitochondria for injection during intracytoplasmic sperm injection (ICSI) [52].

Consistent with the positive benefits reported for heterologous ooplasmic (mitochondrial) transfer in human assisted reproduction, early clinical experience with AUGMENT has been encouraging [115,116]. In 93 IVF patients diagnosed with poor egg and embryo quality at the Toronto Center for Assisted Reproductive Technologies (TCART) in Canada or at FAKIH-IVF in the United Arab Emirates, use of AUGMENT increased clinical pregnancy rates per initiated assisted reproduction cycle by 3–6-fold when compared with matched historical data from prior IVF attempts without AUGMENT in the same patient cohort (25.7% versus 5.2%, respectively) [115,116]. Likewise, live birth rates jumped from historical levels of 1.3% in this patient population to over 18% in the same patient population with just a single cycle of AUGMENT, leading to the birth of 23 babies [115,116] (reviewed in [52]). Using a direct comparative approach termed Matched Best Embryo Selection and Transfer (MBEST), in which eggs retrieved from each patient were allocated to undergo IVF through ICSI or ICSI with AUGMENT, it was further shown that embryo transfer rates were seven-fold higher in the ICSI + AUGMENT group compared to those who underwent ICSI alone [115]. This outcome was attributed directly to dramatic improvements in the selection criteria used to determine if a given blastocyst-stage embryo developed in vitro was suitable for transfer back to the IVF patient. By directly comparing the outcomes obtained using eggs from the same IVF cycle of the same patient, with the only variable being whether AUGMENT was included, these types of studies argue strongly that an exogenous, but autologous, germline mitochondrial boost significantly enhances the post-fertilization developmental competency of human eggs. Continuing studies in animal models have not only supported these observations [117], but have also raised the question of whether mitochondria from germline stem cells or adult stem cells in general are needed to achieve a restoration of egg and embryo quality in aged females [118].

A major variable not to be overlooked here when considering the impact of mitochondrial transfer in these and other studies is the impact of an exogenous mitochondrial boost on eggs in general versus on eggs of specifically reproductively aged females. This can be a major source of confusion if one is not careful in evaluating the published literature on this subject. As just one example, a recent study reported that injection of hepatocyte mitochondria into eggs of young adult gonadotropin-primed female mice collected from the oviducts 14 h (referred to by the authors as fresh) versus 20–24 h (referred to by the authors as in-vivo aged) after induced ovulation failed to affect any aspect of post-IVF preimplantation embryogenesis [119]. At first glance, the outcomes of this study appear at odds with an earlier report that concluded injection of hepatocyte mitochondria into very early zygotes (eggs immediately following fertilization) significantly improves preimplantation embryonic developmental outcomes [114]. However, a key difference in the two reports is that the first studied eggs of only young mice, despite use of the term aged to refer to a group of oocytes in their study, whereas the second focused on zygotes (eggs) of aged mice. It is not surprising that eggs of young adult mice, which already exhibit very high fertilization and embryonic development rates, would be relatively unaffected by an exogenous mitochondrial boost. A similar case can be made for another recent paper concluding from studies using eggs of only young adult mice that injection of mitochondria collected from induced pluripotent stem cells (iPSCs) had no effect on embryonic developmental parameters versus those observed obtained with mock-injected eggs [120].

How exactly does provision of exogenous mitochondria improve egg and embryo quality in females at advanced maternal ages? The prevailing belief is that the injected mitochondria provide an additional source of bioenergetic potential for oocytes impaired in some manner by maternal aging, thus enabling the eggs to regain full functional competency after fertilization. This proposed mode of action would agree with a now large body of evidence linking mitochondrial function and ATP availability to egg and embryo quality across species [34,45,51,79]. However, there are other potential explanations that warrant future investigation. One centers on potential functional differences in mitochondria, or more specifically mitochondrial subtypes, derived from the donor cells when compared with the mitochondrial population present in eggs of aged females, that are outside of the realm of energetics. Notably, in published examples of where an exogenous mitochondrial boost has been reported to improve post-fertilization outcomes in recipient eggs, the donor cells are either from chronologically younger females [104,105,106,107,108,109,110,111] or from stem-like cells that are chronologically the same age as the recipient eggs, from a whole organism perspective, but nonetheless younger from a cellular differentiation perspective [52,114,115,116,118]. If cross-talk between the nucleus and specific mitochondrial subpopulations is disrupted by maternal aging such that mitochondria in eggs become inherently unable to receive or process instructions provided by nuclear-coded gene products trafficked to these organelles, injection of a bolus of non-compromised or responsive donor mitochondria may help restore homeostatic function in eggs needed for embryogenesis to progress successfully. Alternatively, provision of young mitochondria to aged oocytes could directly impact on ROS levels present in the injected eggs, which could then influence signal transduction pathways or ion flux events important for achieving full developmental competency. Rigorous testing of these possibilities would be facilitated by development of new technological platforms that enable analysis and sorting of mitochondrial subtypes based on distinguishing features, such as size, membrane polarization status or biomarkers. Once purified, the mitochondrial subpopulations could then be processed for a range of comparative downstream analyses, including differences in functional properties and proteomic profiles. This information may uncover novel insights into how aging affects mitochondria in germ cells, and how exogenous mitochondria from non-aged sources can restore developmental competency in eggs.

## 5. Mitochondrial Dynamics in Artificial Oocytes

In 2006, Yamanaka and colleagues reported with mice that embryonic and adult fibroblasts could be reprogrammed into iPSCs, a population of embryonic-like pluripotent stem cells with the broad lineage differentiation potential of embryonic stem cells (ESCs) [121]. The following year, the same group reported that a comparable outcome could be obtained using adult human dermal fibroblasts [122]. This breakthrough opened many new possibilities for application of regenerative medicine to combat an array of human health conditions. In numerous studies that have since followed, derivation of most somatic cell lineages from iPSCs has been achieved [123,124], and the first human clinical trials using iPSC-based technologies are underway [125,126,127,128]. Efforts to derive functional germ cells from mouse iPSCs have paralleled those directed at somatic lineage specification [129,130,131], ultimately leading to reconstitution of the entire life cycle of the mouse female germ line in vitro from primordial germ cell-like cells (PGCLCs) derived from iPSCs in culture [132]. These latter studies, coupled with reports of PGCLC specification from human iPSCs [133,134], have sparked considerable discussion over the potential for use of human iPSC-derived oocytes in assisted reproduction for women in the future [135,136,137,138].

It is important to bear in mind, however, that significant questions still surround the use of iPSCs to generate functional eggs, even in mice. For example, the senior author of the most recent effort to do this emphasized that many of the eggs produced entirely ex vivo by mouse iPSCs were of poor quality [136], and only 3.5% of the embryos generated by in vitro-derived artificial eggs gave rise to offspring [132]. Although these problems could and probably do arise from many potential issues associated with attempting to recreate the complexity of mammalian female gametogenesis in a dish—ranging from limitations in reprogrammed somatic cells to form the germ lineage to epigenetic abnormalities and aneuploidy, one key factor is mitochondrial integrity. Indeed, the adult somatic cell population that is reprogrammed (most often differentiated fibroblasts) would harbor mtDNA molecules with various mutational loads accumulated through duplication errors and oxidative damage over the lifespan of that lineage until collection. It has been recently shown that adult fibroblast-derived human iPSCs indeed contain significant amounts of mtDNA mutations [139]. What happens to detrimental mtDNA molecules in adult somatic cells that are first nuclear-reprogrammed into iPSCs and then used to specify PGCLCs that are further manipulated to generate eggs in vitro for fertilization and embryogenesis? One of two possibilities must be occurring: the PGCLCs specified from the reprogrammed adult somatic cells somehow orchestrate an in-vitro version of the in-vivo germline mtDNA bottleneck and purifying selection, or the artificial eggs generated by PGCLCs in-vitro suffer from inadequate selection against detrimental mtDNA mutations being carried over into the next generation.

This question requires experimental resolution before iPSC-based female gamete generation can be even cautiously viewed in the context of translation to humans, with such studies designed to unequivocally determine if an mtDNA bottleneck occurs during in-vitro derivation of germ cells, and eventually eggs, from iPSCs. If an in-vitro bottleneck is not observed, the safety of iPSC-based technologies to produce female gametes for clinical use would be highly questionable. However, if evidence of an in-vitro bottleneck is observed, this model would provide a powerful new means to study mtDNA dynamics and inheritance during germline specification and differentiation under defined conditions in vitro, to compare the efficiencies of in-vitro and in-vivo germline mtDNA bottlenecks, and to more definitively define the developmental window(s) during which the germline mtDNA bottleneck takes place. Moreover, this approach would circumvent the two main obstacles faced by scientists interested in deciphering how different types or subpopulations of mitochondria, and the genomic material contained within them, are managed in the female germ line during the development of mature eggs from primitive precursor germ cells: the limited supply of eggs and embryos available for detailed studies of mitochondrial biology, and the technical difficulties in evaluating PGC development in utero.

## 6. Conclusions and Future Studies

Although numerous reviews of mitochondrial dynamics in female germ cells and early embryos have been published over the years, the discussions above highlight how many significant gaps in knowledge still exist in this critical area of study. The central importance of mitochondrial energetics to both egg and embryo quality has been investigated in detail, and is now considered by most working in the field as well established. However, comparatively little is known of how, or even if, other functions of mitochondria outside of ATP production contribute to the successful orchestration of oocyte maturation and preimplantation embryogenesis. Such information may by key to solving the myriad of questions surrounding the mechanisms by which maternal aging negatively impacts on oocyte and embryo quality, as well as on mtDNA segregation during preimplantation embryogenesis. In evaluating the discussions above, we believe the most significant obstacles faced by those interested in advancing this field, both scientifically and clinically, have been rooted in several fundamental limitations. The first, which is broadly relevant to the study of mitochondria and aging in any cell type or organ system, is technological in nature. Specifically, a platform that enables identification, analysis and sorting of different subpopulations of mitochondria from small biological samples would finally allow detailed comparative studies to be performed on mitochondria viewed as a heterogeneous mixture of organelles that share some properties but also exhibit significant differences in proteomic landscape and function. We expect such studies would reveal that at least some perturbations in mitochondrial function in cells with age reflect alterations confined to specific subtypes of mitochondria, thus enabling a more focused approach to the development of potential therapeutic interventions that target the defective subpopulation rather than the entire mitochondrial pool.

Likewise, there is a pressing need for a DNA sequencing platform that enables accurate identification of mutations in entire individual mtDNA molecules for construction of high-quality molecular phylogenies. If mutations are distributed non-uniformly across mtDNA molecules (i.e., some molecules are more heavily mutated while others are not), then sequencing by conventional short-fragment analysis erases any information about such non-uniformity and merely yields information on the average mutant fraction. This is of critical importance for mtDNA inheritance studies because it is imperative to know if preferential inheritance of molecules with a low mutational profile is occurring. In other words, what is required is a technology that enables the identification, counting and tracking of mutations, molecule by molecule, across entire mitochondrial genomes. Current third-generation sequencing platforms can offer long reads and high-throughput capacity. Although clearly a step forward over conventional sequencing, a major limitation of third-generation sequencing is a relatively high single-pass error rate [140], which would confound efforts to track true mtDNA mutations. Addition of bell adapters to sequencing templates would allow these templates to be read multiple times in a continuous circle, resulting in a highly accurate circular consensus sequence (CCS). The CCS approach, however, is limited to application with short DNA fragments due to constraints on how long the polymerase remains active. Without a way to correct for sequencing and PCR errors, currently available sequencing technologies cannot be used to accurately analyze long molecules from highly heterogeneous DNA mixtures, which is of necessity for detailed studies of mtDNA inheritance. With continued technological improvements in the accuracy of next-generation sequencing on the horizon [141], this barrier to progress on mtDNA tracking will hopefully be overcome soon.

Finally, the recent recapitulation of mouse female gametogenesis, from iPSC-derived PGCLCs to fertilizable eggs and preimplantation embryos (that then yield offspring after embryo transfer into recipient females) [131], entirely in vitro may offer a new means to study mitochondrial dynamics in the female germ line under experimental conditions that can provide larger numbers of germ cells at defined stages for study and that are not constrained by the inherent issues associated with studies of female germ line development in vivo. Furthermore, by reprogramming somatic cells collected at different points during chronological aging of the animal, the resultant iPSC lines may prove useful for assessing the impact of aging on mitochondrial dynamics and function during germline specification and differentiation, and on the eggs and embryos formed from such germ cells. Of course, these types of studies will be contingent upon the outcome of what hopefully will be more near-term assessments of mitochondrial quality-control throughout the entire process of artificial female gametogenesis from iPSCs, including a thorough analysis of mtDNA in offspring. To that end, there is an additional level of complexity that needs to be considered in future studies, which revolves around the fact that the landscape of nuclear-encoded gene products localized in mitochondria vary significantly across cell lineages [11,12,13]. In other words, the mitochondrial proteomic identity of a given cell type is determined by the nuclear identity of that cell. If a differentiated adult somatic cell, such as a fibroblast, is reprogrammed at the nuclear level to become an iPSC that is then differentiated into a germ cell-like cell, is the mitochondrial proteome of the original parent cell reprogrammed in parallel to match the intermediate pluripotent state and, subsequently, the new germ lineage identity? If so, how does aging impact on this intracellular re-wiring process? Currently, we have more questions than answers, but exciting studies are on the horizon that will open further insights into how aging interfaces with mitochondrial heterogeneity, dynamics and function in the female germ line.

## References

[1] Rockstein M., Brandt K.F. (1963). Enzyme changes in flight muscle correlated with aging and flight availability in the male housefly. Science.

[2] Balaban R.S., Nemoto S., Finkel T. (2005). Mitochondria, oxidants, and aging. Cell.

[3] Lee H.-C., Wei Y.-H. (2012). Mitochondria and aging. Adv. Exp. Med. Biol..

[4] Bractic A., Larsson N.-G. (2013). The role of mitochondria in aging. J. Clin. Investig..

[5] Chistiakov D.A., Sobenin I.A., Revin V.V., Orekhov A.N., Bobryshev Y.V. (2014). Mitochondrial aging and age-related dysfunction of mitochondria. BioMed Res. Int..

[6] Gonzalez-Freire M., de Cabo R., Bernier M., Sollott S.J., Fabbri E., Navas P., Ferrucci L. (2015). Reconsidering the role of mitochondria in aging. J. Gerontol. A Biol. Sci. Med. Sci..

[7] Sun N., Youle R.J., Finkel T. (2016). The mitochondrial basis of aging. Mol. Cell.

[8] Kauppila T.E.S., Kauppila J.H.K., Larsson N.-G. (2017). Mammalian mitochondria and aging: An update. Cell Metab..

[9] Marchi S., Bittremieux M., Missiroli S., Morganti C., Patergnani S., Sbano L., Rimessi A., Kerkhofs M., Parys J.B., Bultynck G. (2017). Endoplasmic reticulum-mitochondria communication through Ca^2+^ signaling: The importance of mitochondria-associated membranes (MAMs). Adv. Exp. Med. Biol..

[10] Suofu Y., Li W., Jean-Alphonse F.G., Jia J., Khattar N.K., Li J., Baranov S.V., Leronni D., Mihalik A.C., He Y. (2017). Dual role of mitochondria in producing melatonin and driving GPCR signaling to block cytochrome *c* release. Proc. Natl. Acad. Sci. USA.

[11] Palmfeldt J., Bross P. (2017). Proteomics of human mitochondria. Mitochondrion.

[12] Kruse R., Hojlund H. (2017). Mitochondrial phosphoproteomics of mammalian tissues. Mitochondrion.

[13] Carrico C., Meyer J.G., He W., Gibson B.W., Verdin W. (2018). The mitochondrial acylome emerges: Proteomics, regulation by sirtuins, and metabolic and disease implications. Cell Metab..

[14] Butow R.A., Avadhani N.G. (2004). Mitochondrial signaling: The retrograde response. Mol. Cell.

[15] Guha M., Avadhani N.G. (2013). Mitochondrial retrograde signaling at the crossroads of tumor bioenergetics, genetics and epigenetics. Mitochondrion.

[16] Jazwinski S.M. (2013). The retrograde response: When mitochondrial quality control is not enough. Biochim. Biophys. Acta.

[17] Cagin U., Enriquez J.A. (2015). The complex crosstalk between mitochondria and the nucleus: What goes on in between?. Int. J. Biochem. Cell Biol..

[18] Hutchinson C.A., Newbold J.E., Potter S.S. (1974). Maternal inheritance of mammalian mitochondrial DNA. Nature.

[19] Giles R.E., Blanc H., Cann H.M., Wallace D.C. (1980). Maternal inheritance of human mitochondrial DNA. Proc. Natl. Acad. Sci. USA.

[20] Kaneda H., Hayashi J., Takahama S., Taya C., Lindahl K.F., Yonekawa H. (1995). Elimination of paternal mitochondrial DNA in intraspecific crosses during early mouse embryogenesis. Proc. Natl. Acad. Sci. USA.

[21] Cummins J. (1998). Mitochondrial DNA in mammalian reproduction. Rev. Reprod..

[22] Sutovsky P., Moreno R.D., Ramalho-Santos J., Dominko T., Simerly C., Schatten G. (2000). Ubiquitinated sperm mitochondria, selective proteolysis, and the regulation of mitochondrial inheritance in mammalian embryos. Biol. Reprod..

[23] Sutovsky P., Van Leyen K., McCauley T., Day B.N., Sutovsky M. (2004). Degradation of paternal mitochondria after fertilization: Implications for heteroplasmy, assisted reproductive technologies and mtDNA inheritance. Reprod. Biomed. Online.

[24] Al Rawi S., Louvet-Vallée S., Djeddi A., Sachse M., Culetto E., Hajjar C., Boyd L., Legouis R., Galy V. (2011). Postfertilization autophagy of sperm organelles prevents paternal mitochondrial DNA transmission. Science.

[25] Piko L., Matsumoto L. (1976). Number of mitochondria and some properties of mitochondrial DNA in the mouse egg. Dev. Biol..

[26] Motta P.M., Nottola S.A., Makabe S., Heyn R. (2000). Mitochondrial morphology in human fetal and adult female germ cells. Hum. Reprod..

[27] Jansen R.P., Burton G.J. (2004). Mitochondrial dysfunction in reproduction. Mitochondrion.

[28] Khrapko K., Coller H.A., Andre P.C., Li X.C., Hanekamp J.S., Thilly W.G. (1997). Mitochondrial mutational spectra in human cells and tissues. Proc. Natl. Acad. Sci. USA.

[29] Faddy M.J., Gosden R.G., Gougeon A., Richardson S.J., Nelson J.F. (1992). Accelerated disappearance of ovarian follicles in mid-life: Implications for forecasting menopause. Hum. Reprod..

[30] Szamatowicz M., Grochowski D. (1998). Fertility and infertility in aging women. Gynecol. Endocrinol..

[31] Buckler H. (2005). The menopause transition: Endocrine changes and clinical symptoms. J. Br. Menopause Soc..

[32] Oktem O., Oktay K. (2008). The ovary: Anatomy and function throughout human life. Ann. N. Y. Acad. Sci..

[33] Broekmans F.J., Soules M.R., Fauser B.C. (2009). Ovarian aging: Mechanisms and clinical consequences. Endocr. Rev..

[34] Tilly J.L., Sinclair D.A. (2013). Germline energetics, aging and female infertility. Cell Metab..

[35] Navot D., Bergh P.A., Williams M.A., Garrisi G.J., Guzman I., Sandler B., Grunfeld L. (1991). Poor oocyte quality rather than implantation failure as a cause of age-related decline in female fertility. Lancet.

[36] Sauer M.V., Paulson R.J., Lobo R.A. (1995). Pregnancy in women 50 or more years of age: Outcomes of 22 consecutively established pregnancies from oocyte donation. Fertil. Steril..

[37] Henderson S.A., Edwards R.G. (1968). Chiasma frequency and maternal age in mammals. Nature.

[38] Hook E.B. (1981). Rates of chromosome abnormalities at different maternal ages. Obstet. Gynecol..

[39] Hassold T., Chiu D. (1985). Maternal age-specific rates of numerical chromosome abnormalities with special reference to trisomy. Hum. Genet..

[40] Battaglia D.E., Goodwin P., Klein N.A., Soules M.R. (1996). Influence of maternal age on meiotic spindle assembly in oocytes from naturally cycling women. Hum. Reprod..

[41] Tarín J.J., Pérez-Albalá S., Cano A. (2001). Cellular and morphological traits of oocytes retrieved from aging mice after exogenous ovarian stimulation. Biol. Reprod..

[42] Eichenlaub-Ritter U., Vogt E., Yin H., Gosden R. (2004). Spindles, mitochondria and redox potential in ageing oocytes. Reprod. Biomed. Online.

[43] Pan H., Ma P., Zhu W., Schultz R.M. (2008). Age-associated increase in aneuploidy and changes in gene expression in mouse eggs. Dev. Biol..

[44] Hunt P.A., Hassold T.J. (2008). Human female meiosis: What makes a good egg go bad?. Trends Genet..

[45] Selesniemi K., Lee H.-J., Muhlhauser A., Tilly J.L. (2011). Prevention of maternal aging-associated oocyte aneuploidy and meiotic spindle defects in mice by dietary and genetic strategies. Proc. Natl. Acad. Sci. USA.

[46] Merriman J.A., Jennings P.C., McLaughlin E.A., Jones K.T. (2012). Effect of aging on superovulation efficiency, aneuploidy rates, and sister chromatid cohesion in mice aged up to 15 months. Biol. Reprod..

[47] Fu X., Cheng J., Hou Y., Zhu S. (2014). The association between the oocyte pool and aneuploidy: A comparative study of the reproductive potential of young and aged mice. J. Assist. Reprod. Genet..

[48] Franasiak J.M., Forman E.J., Hong K.H., Werner M.D., Upham K.M., Treff N.R., Scott R.J. (2014). The nature of aneuploidy with increasing age of the female partner: A review of 15,169 consecutive trophectoderm biopsies evaluated with comprehensive chromosomal screening. Fertil. Steril..

[49] Tarín J.J., Pérez-Albalá S., Cano A. (2002). Oral antioxidants counteract the negative effects of female aging on oocyte quantity and quality in the mouse. Mol. Reprod. Dev..

[50] Selesniemi K., Lee H.-J., Tilly J.L. (2008). Moderate caloric restriction initiated in rodents during adulthood sustains function of the female reproductive axis into advanced chronological age. Aging Cell.

[51] Bentov Y., Yavorska T., Esfandiari N., Jurisicova A., Casper R.F. (2011). The contribution of mitochondrial function to reproductive aging. J. Assist. Reprod. Genet..

[52] Woods D.C., Tilly J.L. (2015). Autologous germline mitochondrial energy transfer (AUGMENT) in human assisted reproduction. Semin. Reprod. Med..

[53] Scarpulla R.C. (2002). Transcriptional activators and coactivators in the nuclear control of mitochondrial function in mammalian cells. Gene.

[54] Villena J.A. (2015). New insights into PGC-1 coactivators: Redefining their role in the regulation of mitochondrial function and beyond. FEBS J..

[55] Liu M., Yin Y., Ye X., Zeng M., Zhao Q., Keefe D.L., Liu L. (2013). Resveratrol protects against age-associated infertility in mice. Hum. Reprod..

[56] Ben-Meir A., Burstein E., Borrego-Alvarez A., Chong J., Wong E., Yavorska T., Naranian T., Chi M., Wang Y., Bentov Y. (2015). Coenzyme Q10 restores oocyte mitochondrial function and fertility during reproductive aging. Aging Cell.

[57] Li Y.R., Li S., Lin C.C. (2018). Effect of resveratrol and pterostilbene on aging and longevity. Biofactors.

[58] Hernández-Camacho J.D., Bernier M., López-Lluch G., Navas P. (2018). Coenzyme Q10 supplementation in aging and disease. Front. Physiol..

[59] Keefe D.L., Niven-Fairchild T., Powell S., Buradagunta S. (1995). Mitochondrial deoxyribonucleic acid deletions in oocytes and reproductive aging in women. Fertil. Steril..

[60] Hsieh R.H., Tsai N.M., Chang S.J., Wei Y.H., Tzeng C.R. (2002). Multiple rearrangements of mitochondrial DNA in unfertilized human oocytes. Fertil. Steril..

[61] Chan C.C., Liu V.W., Lau E.Y., Yeung W.S., Ng E.H., Ho P.C. (2005). Mitochondrial DNA content and 4977 bp deletion in unfertilized oocytes. Mol. Hum. Reprod..

[62] Ashley M.V., Laipis P.J., Hauswirth W.W. (1989). Rapid segregation of heteroplasmic bovine mitochondria. Nucl. Acids Res..

[63] Jenuth J.P., Peterson A.C., Fu K., Shoubridge E.A. (1996). Random genetic drift in the female germline explains the rapid segregation of mammalian mitochondrial DNA. Nat. Genet..

[64] Jansen R.P., de Boer K. (1998). The bottleneck: Mitochondrial imperatives in oogenesis and ovarian follicular fate. Mol. Cell. Endocrinol..

[65] Stewart J.B., Freyer C., Elson J.L., Wredenberg A., Cansu Z., Trifunovic A., Larsson N.-G. (2008). Strong purifying selection in transmission of mammalian mitochondrial DNA. PLoS Biol..

[66] Wai T., Teoli D., Shoubridge E.A. (2008). The mitochondrial DNA genetic bottleneck results from replication of a subpopulation of genomes. Nat. Genet..

[67] Cree L.M., Samuels D.C., de Sousa Lopes S.C., Rajasimha H.K., Wonnapinij P., Mann J.R., Dahl H.H., Chinnery P.F. (2008). A reduction of mitochondrial DNA molecules during embryogenesis explains the rapid segregation of genotypes. Nat. Genet..

[68] Schon E.A., DiMauro S., Hirano M. (2012). Human mitochondrial DNA: Roles of inherited and somatic mutations. Nat. Rev. Genet..

[69] Rebolledo-Jaramillo B., Su M.S.-W., Stoler N., McElhoe J.A., Dickins B., Blankenberg D., Korneliussen T.S., Chiaromonte F., Nielsen R., Holland M.M. (2014). Maternal age effect and severe germ-line bottleneck in the inheritance of human mitochondrial DNA. Proc. Natl. Acad. Sci. USA.

[70] Stewart J.B., Larsson N.-G. (2014). Keeping mtDNA in shape between generations. PLoS Genet..

[71] Stewart J.B., Chinnery P.F. (2015). The dynamics of mitochondrial DNA heteroplasmy: Implications for human health and disease. Nat. Rev. Genet..

[72] Floros V.I., Pyle A., Dietmann S., Wei W., Tang W.C., Irie N., Payne B., Capalbo A., Noli L., Coxhead J. (2018). Segregation of mitochondrial DNA heteroplasmy through a developmental genetic bottleneck in human embryos. Nat. Cell Biol..

[73] Cao L., Shitara H., Horii T., Nagao Y., Imai H., Abe K., Hara T., Hayashi J., Yonekawa H. (2007). The mitochondrial bottleneck occurs without reduction of mtDNA content in female mouse germ cells. Nat. Genet..

[74] Samuels D.C., Wonnapinij P., Cree L.M., Chinnery P.F. (2010). Reassessing evidence for a postnatal mitochondrial genetic bottleneck. Nat. Genet..

[75] Wai T., Shoubridge E.A. (2010). Reply: Reassessing evidence for a postnatal mitochondrial genetic bottleneck. Nat. Genet..

[76] Lee H.S., Ma H., Juanes R.C., Tachibana M., Sparman M., Woodward J., Ramsey C., Xu J., Kang E.J., Amato P. (2012). Rapid mitochondrial DNA segregation in primate preimplantation embryos precedes somatic and germline bottleneck. Cell Rep..

[77] Ross J.M., Stewart J.B., Hagström E., Brené S., Mourier A., Coppotelli G., Freyer C., Lagouge M., Hoffer B.J., Olson L. (2013). Germline mitochondrial DNA mutations aggravate ageing and can impair brain development. Nature.

[78] Ross J.M., Coppotelli G., Hoffer B.J., Olson L. (2014). Maternally transmitted mitochondrial DNA mutations can reduce lifespan. Sci. Rep..

[79] Van Blerkom J. (2004). Mitochondria in human oogenesis and preimplantation embryogenesis: Engines of metabolism, ionic regulation and developmental competence. Reproduction.

[80] Van Blerkom J., Davis P., Mathwig V., Alexander S. (2002). Domains of high-polarized and low-polarized mitochondria may occur in mouse and human oocytes and early embryos. Hum. Reprod..

[81] Van Blerkom J., Davis P., Alexander S. (2003). Inner mitochondrial membrane potential (ΔΨm), cytoplasmic ATP content and free Ca^2+^ levels in metaphase II mouse oocytes. Hum. Reprod..

[82] Hashimoto S., Morimoto N., Yamanaka M., Matsumoto H., Yamochi T., Goto H., Inoue M., Nakaoka Y., Shibahara H., Morimoto Y. (2017). Quantitative and qualitative changes of mitochondria in human preimplantation embryos. J. Assist. Reprod. Genet..

[83] Graham S.J., Zernicka-Goetz M. (2016). The acquisition of cell fate in mouse development: How do cells first become heterogeneous?. Curr. Top. Dev. Biol..

[84] Mihajlovic A.I., Bruce A.W. (2017). The first cell-fate decision of mouse preimplantation embryo development: Integrating cell position and polarity. Open Biol..

[85] Mattson M.P., Partin J. (1999). Evidence for mitochondrial control of neuronal polarity. J. Neurosci. Res..

[86] Fu D., Mitra K., Sengupta P., Jarnik M., Lippincott-Schwartz J., Arias I.M. (2013). Coordinated elevation of mitochondrial oxidative phosphorylation and autophagy help drive hepatocyte polarity. Proc. Natl. Acad. Sci. USA.

[87] Diaz G., Setzu H., Zucca A., Isola R., Diana A., Murru R., Sogos V., Gremo F. (1999). Subcellular heterogeneity of mitochondrial membrane potential: Relationship with organelle distribution and intercellular contacts in normal, hypoxic and apoptotic cells. J. Cell Sci..

[88] Reynier P., May-Panloup P., Chrétien M.F., Morgan C.J., Jean M., Savagner F., Barrière P., Malthièry Y. (2001). Mitochondrial DNA content affects the fertilizability of human oocytes. Mol. Hum. Reprod..

[89] May-Panloup P., Chrétien M.F., Jacques C., Vasseur C., Malthièry Y., Reynier P. (2005). Low oocyte mitochondrial DNA content in ovarian insufficiency. Hum. Reprod..

[90] Santos T.A., El Shourbagy S., St John J.C. (2006). Mitochondrial content reflects oocyte variability and fertilization outcome. Fertil. Steril..

[91] Duran H.E., Simsek-Duran F., Oehninger S.C., Jones H.W., Castora F.J. (2011). The association of reproductive senescence with mitochondrial quantity, function, and DNA integrity in human oocytes at different stages of maturation. Fertil. Steril..

[92] Murakoshi Y., Sueoka K., Takahashi K., Sato S., Sakurai T., Tajima H., Yoshimura Y. (2013). Embryo developmental capability and pregnancy outcome are related to the mitochondrial DNA copy number and ooplasmic volume. J. Assist. Reprod. Genet..

[93] Simsek-Duran F., Li F., Ford W., Swanson R.J., Jones H.W. Jr., Castora F.J. (2013). Age-associated metabolic and morphologic changes in mitochondria of individual mouse and hamster oocytes. PLoS ONE.

[94] Larrson N., Wang J., Wilhemsson H., Oldfors A., Rustin P., Lewandoski M., Barsh G., Clayton D. (1998). Mitochondria transcription factor A is necessary for mtDNA maintenance and embryogenesis in mice. Nat. Genet..

[95] Thundathil J., Filion F., Smith L.C. (2005). Molecular control of mitochondrial function in preimplantation mouse embryos. Mol. Reprod. Dev..

[96] Boudoures A.L., Saben J., Drury A., Scheaffer S., Modi Z., Zhang W., Moley K.H. (2017). Obesity-exposed oocytes accumulate and transmit damaged mitochondria due to an inability to activate mitophagy. Dev. Biol..

[97] Tsukamoto S., Kuma A., Mizushima N. (2008). The role of autophagy during the oocyte-to-embryo transition. Autophagy.

[98] Lee G.K., Shin H., Lim H.J. (2016). Rapamycin influences the efficiency of in vitro fertilization and development in the mouse: A role for autophagic activation. Asian-Australas. J. Anim. Sci..

[99] Fragouli E., Spath K., Alfarawati S., Kaper F., Craig A., Michel C.E., Kokocinski F., Cohen J., Munné S., Wells D. (2015). Altered levels of mitochondrial DNA are associated with female age, aneuploidy, and provide an independent measure of embryonic implantation potential. PLoS Genet..

[100] Kushnir V.A., Ludaway T., Russ R.B., Fields E.J., Koczor C., Lewis W. (2012). Reproductive aging is associated with decreased mitochondrial abundance and altered structure in murine oocytes. J. Assist. Reprod. Genet..

[101] Ravichandran K., McCaffrey C., Grifo J., Morales A., Perloe M., Munné S., Wells D., Fragouli E. (2017). Mitochondrial DNA quantification as a tool for embryo viability assessment: Retrospective analysis of data from single euploid transfers. Hum. Reprod..

[102] Fragouli E., McCaffrey C., Ravichandran K., Spath K., Grifo J.A., Munné S., Wells D. (2017). Clinical implications of mitochondrial DNA quantification on pregnancy outcomes: A blinded prospective non-selection study. Hum. Reprod..

[103] Leese H.J. (2002). Quiet please, do not disturb: A hypothesis of embryo metabolism and viability. Bioessays.

[104] Cohen J., Scott R., Schimmel T., Levron J., Willadsen S. (1997). Birth of an infant after transfer of anucleate donor oocyte cytoplasm into recipient eggs. Lancet.

[105] Cohen J., Scott R., Alikani M., Schimmel T., Munné S., Levron J., Wu L., Brenner C.A., Warner C., Willadsen S. (1998). Ooplasmic transfer in mature human oocytes. Mol. Hum. Reprod..

[106] Lazendorf S.E., Mayer J.F., Toner J., Oehninger S., Saffan D.S., Muasher S. (1999). Pregnancy following transfer of ooplasm from cryopreserved-thawed donor oocytes into recipient oocytes. Fertil. Steril..

[107] Huang C.C., Cheng T.C., Chang H.H., Chang C.C., Chen C.I., Liu J., Lee M.S. (1999). Birth after the injection of sperm and the cytoplasm of tripronucleate zygotes into metaphase II oocytes in patients with repeated implantation failure after assisted fertilization procedures. Fertil. Steril..

[108] Dale B., Wilding M., Botta G., Rasile M., Marino M., Di Matteo L., De Placido G., Izzo A. (2001). Pregnancy after cytoplasmic transfer in a couple suffering from idiopathic infertility: Case report. Hum. Reprod..

[109] Barritt J.A., Brenner C.A., Malter H.E., Cohen J. (2001). Mitochondria in human offspring derived from ooplasmic transplantation. Hum. Reprod..

[110] Brenner C.A., Barritt J.A., Willadsen S., Cohen J. (2000). Mitochondrial DNA heteroplasmy after human ooplasmic transplantation. Fertil. Steril..

[111] Barritt J., Willadsen S., Brenner C., Cohen J. (2001). Cytoplasmic transfer in assisted reproduction. Hum. Reprod. Update.

[112] Zoon K.C. Human cells used in therapy involving the transfer of genetic material by means other than the union of gamete nuclei. https://www.fda.gov/BiologicsBloodVaccines/CellularGeneTherapyProducts/ucm2007205.htm.

[113] El Shourbagy S.H., Spikings E.C., Freitas M., St John J.C. (2006). Mitochondria directly influence fertilisation outcome in the pig. Reproduction.

[114] Yi Y.C., Chen M.J., Ho J.Y.P., Guu H.F., Ho E.S. (2007). Mitochondrial transfer can enhance the murine embryo development. J. Assist. Reprod. Genet..

[115] Fakih M.H., El Shmoury M., Szeptycki J., dela Cruz D.B., Lux C., Verjee S., Burgess C.M., Cohn G.M., Casper R.F. (2015). The AUGMENT^SM^ treatment: Physician reported outcomes of the initial global patient experience. JFIV Reprod. Med. Genet..

[116] Oktay K., Baltaci V., Sonmezer M., Turan V., Unsal E., Baltaci A., Aktuna S., Moy F. (2015). Oogonial precursor cell-derived autologous mitochondria injection (AMI) to improve outcomes in women with multiple IVF failures due to low oocyte quality: A clinical translation. Reprod. Sci..

[117] Cagnone G.L., Tsai T.S., Makanji Y., Matthews P., Gould J., Bonkowski M.S., Elgass K.D., Wong A.S., Wu L.E., St John J.C. (2016). Restoration of normal embryogenesis by mitochondrial supplementation in pig oocytes exhibiting mitochondrial DNA deficiency. Sci. Rep..

[118] Wang Z.-B., Hao J.-X., Meng T.-G., Guo L., Dong M.-Z., Fan L.-H., Ouyang Y.-C., Wang G., Sun Q.-Y., Ou X.-H. (2017). Transfer of autologous mitochondria from adipose tissue-derived stem cells rescues oocyte quality and infertility in aged mice. Aging.

[119] Igarashi H., Takahashi T., Abe H., Nakano H., Nakajima O., Nagase S. (2016). Poor embryo development in post-ovulatory in vivo-aged mouse oocytes is associated with mitochondrial dysfunction, but mitochondrial transfer from somatic cells is not sufficient for rejuvenation. Hum. Reprod..

[120] Li R., Wen B., Zhao H., Ouyang N., Ou S., Wang W., Han J., Yang D. (2017). Embryo development after mitochondrial supplementation from induced pluripotent stem cells. J. Assist. Reprod. Genet..

[121] Takahashi K., Yamanaka S. (2006). Induction of pluripotent stem cells from mouse embryonic and adult fibroblast cultures by defined factors. Cell.

[122] Takahashi K., Tanabe K., Ohnuki M., Narita M., Ichisaka T., Tomoda K., Yamanaka S. (2007). Induction of pluripotent stem cells from adult human fibroblasts by defined factors. Cell.

[123] Cherry A.B.C., Daley G.Q. (2012). Reprogramming and cellular identity for regenerative medicine. Cell.

[124] Takahashi K., Yamanaka S. (2016). A decade of transcription factor-mediated reprogramming to pluripotency. Nat. Rev. Mol. Cell Biol..

[125] Cyranoski D. (2014). Japanese woman is first recipient of next-generation stem cells. Nature.

[126] Cyranoski D. (2015). Stem cells cruise to clinic: Japanese study of induced pluripotent stem cells aims to demonstrate safety in humans. Nature.

[127] Mandai M., Watanabe A., Kurimoto Y., Hirami Y., Morinaga C., Daimon T., Fujihara M., Akimaru H., Sakai N., Shibata Y. (2017). Autologous induced stem-cell–derived retinal cells for macular degeneration. N. Engl. J. Med..

[128] Cyranoski D. (2017). Japanese man is first to receive ‘reprogrammed’ stem cells from another person. Nature.

[129] Hayashi K., Ohta H., Kurimoto K., Aramaki S., Saitou M. (2011). Reconstitution of the mouse germ cell specification pathway in culture by pluripotent stem cells. Cell.

[130] Hayashi K., Ogushi S., Kurimoto K., Shimamoto S., Ohta H., Saitou M. (2012). Offspring from oocytes derived from in vitro primordial germ cell-like cells in mice. Science.

[131] Zhou Q., Wang M., Wang X., Fu R., Wan H., Xie M., Liu M., Guo X., Zheng Y., Feng G. (2016). Complete meiosis from embryonic stem cell-derived germ cells in vitro. Cell Stem Cell.

[132] Hikabe O., Hamazaki N., Nagamatsu G., Obata Y., Hirao Y., Hamada N., Shimamoto S., Imamura T., Nakashima K., Saitou M. (2016). Reconstitution in vitro of the entire cycle of the mouse female germ line. Nature.

[133] Panula S., Medrano J.V., Kee K., Bergström R., Nguyen H.N., Byers B., Wilson K.D., Wu J.C., Simon C., Hovatta O. (2011). Human germ cell differentiation from fetal- and adult-derived induced pluripotent stem cells. Hum. Mol. Genet..

[134] Irie N., Weinberger L., Tang W.W.C., Kobayashi T., Viukov S., Manor Y.S., Dietman S., Hanna J.H., Surani M.A. (2015). SOX17 is a critical specifier of human primordial germ cell fate. Cell.

[135] Cyranoski D. (2014). Rudimentary egg and sperm cells made from stem cells. Nature.

[136] Cyranoski D. (2016). Mouse eggs made from skin cells in a dish. Nature.

[137] Cohen I.G., Daley G.Q., Adashi E.Y. (2017). Disruptive reproductive technologies. Sci. Transl. Med..

[138] Smith J.E.J., Gilchrist R.B. (2016). Are human oocytes from stem cells next?. Nat. Biotechnol..

[139] Kang E., Wang X., Tippner-Hedges R., Ma H., Folmes C., Gutierrez N.M., Lee Y., Van Dyken C., Ahmed R., Li Y. (2016). Age-Related Accumulation of Somatic Mitochondrial DNA Mutations in Adult-Derived Human iPSCs. Cell Stem Cell.

[140] Jiao X., Zheng X., Ma L., Kutty G., Gogineni E., Sun Q., Sherman B.T., Hu X., Jones K., Raley C. (2013). A benchmark study on error assessment and quality control of CCS reads derived from the PacBio RS. J. Data Min. Genom. Proteom..

[141] Salk J.J., Schmitt M.W., Loeb L.A. (2018). Enhancing the accuracy of next-generation sequencing for detecting rare and subclonal mutations. Nat. Rev. Genet..

